# Rhabdomyolysis in a 9-year-old Syrian child due to homozygous nonsense variant in *LPIN1*: A case report of a new pathogenic variant

**DOI:** 10.1097/MD.0000000000051309

**Published:** 2026-10-30

**Authors:** Sultaneh Haddad, Hana Diab, Hanan H. Abunada, Aya Saibouh, Dina Aldebs, Basheer Khalil

**Affiliations:** a Department of Pediatrics, Children’s University Hospital, Damascus, Syrian Arab Republic; b Faculty of Medicine, Beirut Arab University, Beirut, Lebanon; c Biomedical Research Center, QU Health, Qatar University, Doha, Qatar; d Department of Internal Medicine, Damascus University, Faculty of Medicine, Damascus, Syrian Arab Republic; e Rheumatology Department, Children’s University Hospital, Damascus, Syrian Arab Republic.

**Keywords:** genetic testing, *LPIN1*, myoglobinuria, nonsense variant, pediatric, rhabdomyolysis

## Abstract

**Rationale:**

Rhabdomyolysis is a life-threatening condition characterized by acute skeletal muscle breakdown, leading to the release of creatine kinase and myoglobin into the bloodstream. Mutations in the *LPIN1* gene, which encodes lipin-1 (a key enzyme involved in lipid biosynthesis and fatty acid oxidation), represent a leading genetic cause of recurrent rhabdomyolysis in children.

**Patient concerns:**

A 9-year-old Syrian child with no significant family medical history presented with more than 17 episodes of rhabdomyolysis since the age of 2. The episodes were characterized by severe frontal headaches, vomiting, dark red urine, distal muscle weakness, and fever. The current episode had lasted for more than 2 weeks.

**Diagnoses:**

Whole-exome sequencing identified a novel homozygous pathogenic variant in the *LPIN1* gene (c.2362C>T, p.Gln788*).

**Interventions:**

Supportive therapies including taking rest, intravenous fluid, and nutrition support were started.

**Outcomes:**

*LPIN1* mutations account for approximately 60% of pediatric recurrent rhabdomyolysis cases in Western cohorts, driven by impaired fatty acid oxidation and mitochondrial dysfunction under catabolic stress. This case highlights a novel variant associated with severe, recurrent episodes and potential systemic complications, including acute kidney injury. The identification of this novel *LPIN1* variant expands the known mutational spectrum of pediatric rhabdomyolysis.

**Lessons:**

This case underscores the critical role of genetic testing in the diagnosis of recurrent rhabdomyolysis. Early genetic confirmation enables timely intervention and the implementation of preventive strategies to reduce the risk of life-threatening complications.

## 1. Introduction

Rhabdomyolysis (RML) is a fatal disorder caused by sudden skeletal muscle breakdown, allowing enzymes and intracellular proteins such as creatine kinase (CK) and myoglobin to enter the bloodstream.^[1]^ Although *LPIN1* mutations are uncommon in children, they are now recognized as one of the primary genetic causes of recurrent RML, which is becoming more linked to hereditary metabolic myopathies.^[2,3]^ Both males and females are equally affected by the illness, which typically manifests in early childhood, frequently before the age of 6. However, in certain areas, its frequency may be underreported because genetic testing is not readily available.^[3]^ An enzyme called lipin-1, which is encoded by *LPIN1*, plays 2 functions in lipid biosynthesis and fatty acid oxidation. Inadequate energy metabolism can lead to times of muscle degradation following catabolic stressors such as febrile diseases, intense physical exercise, or prolonged fasting.^[2,4]^

Affected children often show signs of myoglobinuria, myalgia, muscle weakness, and a significant increase in CK during crises. Recurrent episodes commonly assist in the diagnosis, which can be confirmed through targeted *LPIN1* sequencing or whole-exome sequencing. Differential diagnoses may include fatty acid oxidation disorders, glycogen storage diseases, mitochondrial myopathies, and inflammatory muscle disorders.^[3,5]^ There is currently no proven cure; management is supportive and includes vigorous intravenous hydration, electrolyte monitoring, and avoiding catabolic triggers. There are also growing reports of corticosteroid use during emergencies.^[2,4]^ The complications include arrhythmias, compartment syndrome, acute renal damage, and electrolyte abnormalities such as hyperkalemia, all of which can be fatal if ignored.^[1]^

## 2. Case presentation

A 9-year-old Syrian male child presented to the clinic with a history of recurrent episodes since age 2, experiencing over 17 episodes. These episodes are characterized by severe frontal headaches, intense vomiting unrelated to food intake, dark red urine, and significant distal muscle weakness not associated with walking or movement. The episodes typically onset suddenly and last 3 to 4 days, though the current episode has persisted for over 2 weeks and is accompanied by fever. Physical exertion was noted to be the trigger of these episodes.

The child has no other medical history or relevant family history.

Physical examination was remarkable for pallor and myalgia. Laboratory investigations revealed significantly elevated levels of CK, erythrocyte sedimentation rate, lactate dehydrogenase, creatinine, alanine aminotransferase, C-reactive protein, and aldolase. The direct Coombs test was negative. Serum biochemical test results are detailed in Table 1.

**Table 1:** Serum biochemical test results.

WBC	HB	MCV	MCHC	Platelets	ESR	CK	Aldolase	Creatinine	LDH	C3
7530 (L: 21%; N: 73%)	13.5	75	36	282,000	53	>46,560	1278	1.35	563	132

CK = creatine kinase, ESR = erythrocyte sedimentation rate, HB = haemoglobin, L = lymphocytes, MCHC = mean corpuscular hemoglobin concentration, MCV = mean corpuscular volume, N = neutrophils, WBC = whole blood count.

Given the recurrent episodes of RML, further genetic testing was pursued. Whole-exome sequencing followed by direct sequencing of the *LPIN1* gene identified a homozygous pathogenic variant in the *LPIN1* gene in the child. Specifically, the *LPIN1* variant c.2362C>T p.(Gln788*) introduces a premature stop codon in exon 18 (of 22). This variant is predicted to trigger nonsense-mediated decay, leading to loss of function, a known disease mechanism for *LPIN1*-related disorders. To our knowledge, this is a novel variant, not previously reported in the literature, and is classified as pathogenic according to CENTOGENE’s implementation of the ACMG/AMP/ClinGen SVI guidelines.

Supportive therapies, including rest, intravenous fluid, and nutrition support, were started, and the CK, alanine aminotransferase, and lactate dehydrogenase had gradually improved. The urine turned clear. Urine myoglobin and hemoglobin testing were negative.

## 3. Discussion

RML is a critical, severe, and potentially life-threatening condition resulting from acute skeletal muscle breakdown. It is characterized by serum creatine kinase elevations exceeding 5 times the upper reference limit, followed by a rapid decline along with myoglobinuria, electrolyte imbalances, and an increased risk of acute renal injury.^[5]^ While fever, viral infections, and intense physical activities are known triggers of RML in children, the underlying genetic defects and metabolic abnormalities significantly increase severity and recurrence risk.^[3]^ Studies have reported that phosphatidate phosphatase-1 (lipin-1) deficiency is associated with episodes of RML. Children under the age of 5 years with *LPIN1* deficiency are at high risk for recurrent RML, underscoring the importance of early clinical recognition and genetic evaluation in this population. Epidemiologically, *LPIN1* mutations account for approximately 60% of pediatric recurrent RML cases in Western cohorts, highlighting its critical role in early-onset illness.^[6]^ The underlying process is caused by loss-of-function mutations in *LPIN1* which encodes lipin-1, a phosphatidic acid phosphatase crucial for mitochondrial function, fatty acid oxidation, and lipid metabolism. Deficiency of lipin-1 leads to mitochondrial dysfunction, impaired fatty acid oxidation, and decreased lipid metabolism, influencing muscle fibers, especially under catabolic stress such as febrile illness or anesthesia.^[7]^ Clinically, children with *LPIN1* deficiency often present with acute episodes of RML: tenderness or muscle pain, general weakness, and dark-colored urine due to myoglobinuria, and markedly elevated serum creatine kinase.^[6]^ These episodes may rapidly progress to systemic manifestations, including fever, vomiting, and electrolyte imbalances with potential acute kidney injury or multi-organ complications if not promptly managed.^[8–10]^ Importantly, between episodes, many patients return to baseline CK levels, although other patients demonstrate persistent mild myopathy, neuropathy, or residual weakness on follow-up, which may delay definitive diagnosis and genetic evaluation.^[11]^ Recognition of *LPIN1*-associated RML is therefore critical for developing preventive strategies and prompt early intervention during acute conditions, taking into consideration that mortality has been reported in children with RML complicated by metabolic crises.

We conducted a literature search of the PubMed database using filters for English-language case reports. The search used the keyword (*LPIN1*). We excluded irrelevant articles and those that were inaccessible, and ultimately reviewed 8 articles.^[3,5,6,12–15]^ We summarized the characteristics of these articles in Table 2.

**Table 2:** Literature review with similar cases of *LPIN1* mutation.

Mutation and inheritance	Age of onset	Symptoms	Treatment	Triggers	Reference
Missense variant c.2047A>G(p.I683V) and in-frame variant c.2107_2108 insAGG(p.Q703delin sQE)	A 14-year-old boy	Intermittent fever, mainly a moderate-to-high fever, low back pain, calf pain	Oral sodium bicarbonate to alkalize urine, fructose and vitamin C to improve metabolism and promote cell repair	Strenuous exercise	Tong and Yu (2021)^[3]^
A homozygous likely pathogenic variant c.1684G>*T* encoding p.(Glu562*) in the *LPIN1* gene	A 1-year-old female	Recurrent myalgia, weakness, dark coloured urine	High calorie diet with extra carbohydrates	Exercise	Indika et al (2020)^[5]^
Compound heterozygous variants (p.Arg388* and p.Arg810Cys) in the *LPIN1* gene	A 9-year-old boy	Cola-colored urine, muscle weakness, and tenderness	Fluid therapy and nutritional support	Exercise	Che et al (2020)^[6]^
Common homozygous 1.7 kb intragenic deletion in *LPIN1*	A 3-year-old boy	Progressive myalgia and emesis loss of energy, delirium, drooling, respiratory distress	IV 10% dextrose-normal saline at 1.5 times maintenance rate, IV dexamethasone at 0.6 mg/kg/d	Gastroenteritis, upper respiratory tract infection	Yeganeh et al (2023)^[12]^
Pathogenic splice site mutation (c.2513 + 1G>A) in the patient and his mother but none in the father	A 11-month-old male infant	Recurrent vomiting, mild diarrhea	Volume substitution with 50 mL/kg crystalloids and normal saline	Not applicable	Mand et al (2023)^[13]^
Homozygous in-frame deletion of exon 18 (c.2295-865_2410-30del) in *LPIN1* The same mutations in the 2 cases	11-year-old girl and 40-year-old female	Case 1: diffuse muscle pain, swelling, and brown coloured urine Case 2: muscle pain and diffuse muscle weakness	Intravenous fluids	Parvovirus infection and metabolic stress, respectively	Pizzamiglio et al (2020)^[14]^
Compound heterozygous variants in *LPIN1*: a pathogenic intragenic deletion involving exon 18 and a likely pathogenic missense variant in exon 19, c.21410G>A, p.(Asp804Asn)	A 46-year-old man	Myalgia	Intravenous fluids and closely monitored for acute kidney injury, cardiac arrhythmias, and electrolyte disturbances	Exercise	Minton et al (2020)^[15]^

## 4. Conclusion

The detection of this novel variant enlarges the mutational spectrum associated with RML and provides more evidence of the gene’s involvement in disease pathogenesis. It emphasizes the importance of genetic testing in pediatrics with RML to provide accurate diagnosis and early preventive strategies.

## Author contributions

**Supervision:** Basheer Khalil.

**Writing – original draft:** Sultaneh Haddad, Hana Diab, Hanan H. Abunada.

**Writing – review & editing:** Aya Saibouh, Dina Aldebs.
